# 

**Published:** 1905-05

**Authors:** 


### BOOKS RECEIVED.

Lea's Series of Medical Epitomes. Edited by Victor C. Pedersen, M. D. Alling and Griffin's Diseases of the Eye and Ear. A Manual for Students and Physicians. By Arthur N. Alling, M. D., Clinical Professor of Ophthalmology in Yale University, and Ovidus Arthur Griffin, B. S., M. D. Duodecimo, 263 pages, with 83 illustrations. Philadelphia and New York: Lea Brothers & Co. ($1.00 net.)

The Johns Hopkins Hospital Reports. Vol. XII. Baltimore: The Johns Hopkins Press. 1904.

The Development of the Human Body. A Manual of Human Embryology. By J. Playfair McMurrich, A. M., Ph. D., Professor of Anatomy in the University of Michigan. Second edition, revised and enlarged. Duodecimo, pp. 539. With 272 illustrations. Philadelphia: P. Blakiston's Son & Co. 1904. (Price, $3.00.)

Medical Philology. Gathered by L. M. Griffiths, M.R.C.S., Eng. Part I. A-EI. Bristol: J. W. Arrowsmith. 1905.

A Handbook of Nursing. For Hospital and General Use. Published under the Direction of the Connecticut Training School for Nurses. Duodecimo, pp. 319. Philadelphia and London: J. B. Lippincott Co. 1905.

Malformations of the Genital Organs of Women. By Ch. Debierre, Professor of Anatomy in the Medical Faculty at Lille. Duodecimo, pp. 182. With 85 illustrations. Translated by J. Henry C. Simes, M. D., Philadelphia: P. Blakiston's Son & Co. 1905. (Price, $1.50).

The Eye, Mind, Energy and Matter. By Chalmers Prentice, M. D. Duodecimo, pp. 131. Chicago: Published by the Author. 1905.

Chemical and Microscopical Diagnosis. By Francis Carter Wood, M. D., Adjunct Professor of Clinical Pathology, Columbia University, New York. Octavo, pp. xxiv-745. With 188 illustrations and 9 colored plates. New York and London: D. Appleton & Co. 1905.

International Clinics. A Quarterly of Illustrated Clinical Lectures and especially prepared articles on Treatment, Medicine, Surgery, Neurology, Pediatrics, Obstetrics, Gynecology, Orthopedics, Pathology, Dermatology, Ophthalmology, Otology, Rhinology, Laryngology, Hygiene and other topics of interest to students and practitioners. By leading members of the medical profession throughout the world. Edited by A. O. J. Kelly, A.M., M.D., Philadelphia. Volume I. Fifteenth series. 1905. Philadelphia and London: J. B. Lippincott Company. (Cloth, $2.00.)

American Edition of Nothnagel's Practice. Diseases of the Blood (Anemia, Chlorosis, Leukemia, Pseudoleukemia). By Dr. P. Ehrlich, Frankfort am Main; Dr. A. Lazarus, Charlottenburg; Dr. K. von Noorden, Frankfort am Main; and Dr. Felix Pinkus, Berlin. Edited, with additions, by Alfred Stengel, M.D., Professor of Clinical Medicine, University of Pennsylvania. Octavo volume of 714 pages, fully illustrated. Philadelphia and London: W. B. Saunders & Company. 1905. (Cloth, $5.00 net; half morocco, $6.00 net.)

A Textbook of Medical Chemistry and Toxicology. By James W. Holland, M.D., Professor of Medical Chemistry and Toxicology, and Dean, Jefferson Medical College, Philadelphia. Octavo volume of 600 pages, fully illustrated, including 8 plates in colors. Philadelphia and London: W. B. Saunders & Company. 1905. (Cloth, $3.00 net.)

Report of the Commissioner of Education for the year 1903. Volume I. Washington: Government Printing Office. 1905.

Mechanical Vibration and Its Therapeutic Application. By M. L. H. Arnold Snow, M.D., Professor of Mechanical Vibration Therapy in the New York School of Physical Therapeutics. Octavo, pp. 297. Illustrated. New York: The Scientific Authors' Publishing Company. 1904. (Price, $2.50.)

